# Linking circulating chemerin and adiponectin to lipoprotein subclasses measured by ^1^H-NMR spectroscopy in 3199 participants from the study of health in Pomerania

**DOI:** 10.1038/s41366-026-02138-8

**Published:** 2026-06-26

**Authors:** Benjamin Spindler, Anke Hannemann, Henry Völzke, Stefan Groß, Martin Bahls, Kathrin Budde, Astrid Petersmann, Ann-Kristin Henning, Theresa Winter, Stephanie Könemann, Marcus Dörr, Matthias Nauck, Nele Friedrich, Stephanie Zylla

**Affiliations:** 1 Institute of Clinical Chemistry and Laboratory Medicine, University Medicine Greifswald, Greifswald, Germany; 2 DZHK (German Centre for Cardiovascular Research), partnersite North, Greifswald, Germany; 3 Institute for Community Medicine, University Medicine Greifswald, Greifswald, Germany; 4 Department of Internal Medicine B, University Medicine Greifswald, Greifswald, Germany; 5 Institute for Clinical Chemistry and Laboratory Medicine, University Medicine Oldenburg, Oldenburg, Germany

**Keywords:** Epidemiology, Obesity, Risk factors, Cardiovascular diseases

## Abstract

**Background/Objectives:**

Associations between adiponectin and chemerin with the most clinically established lipid parameters for assessing cardiovascular risk - LDL-cholesterol, HDL-cholesterol, and triglycerides—are well documented. Since lipoproteins are heterogeneous with respect to size, density, and chemical composition, the examination of their subclasses could help to elucidate the complex interactions between adipokines and lipid metabolism.

**Subjects/Methods:**

Lipoprotein subclasses were quantified by nuclear magnetic resonance spectroscopy in samples from 3199 participants of the Study of Health in Pomerania (SHIP-TREND-0). Associations between adiponectin/chemerin and lipoprotein subclasses were analyzed using appropriately adjusted linear regression models.

**Results:**

The analyses revealed a wide range of statistically significant associations between adiponectin/chemerin and lipoprotein subclasses, that were primarily in opposite directions. Higher adiponectin concentrations were, for example, related to a lower particle number and a lower cholesterol, phospholipid and triglyceride content in atherogenic small, dense LDL-particles, while positive associations were observed for chemerin.

**Conclusions:**

Our highly consistent results demonstrate that high adiponectin was associated with a favorable, anti-atherogenic lipoprotein profile, whereas the opposite was observed for chemerin. Whether these cross-sectional associations reflect causal mechanisms or alternatively, shared underlying metabolic processes, must be clarified by experimental and longitudinal research.

## Introduction

Over the past 50 years, the prevalence of obesity has increased worldwide [1]. It affects whole-body metabolism and contributes to major chronic diseases, including atherosclerosis. Adipose tissue releases multiple bioactive proteins, named adipokines, which affect various metabolic, vascular, and central systems [2]. Here, we focused on two adipokines with opposing effects on the development of atherosclerotic cardiovascular disease (ASCVD), adiponectin and chemerin.

Adiponectin exerts anti-inflammatory effects, inhibits activation of macrophages and endothelial cells, prevents lipid droplet accumulation as well as cholesterol loading in macrophages [3]. Chemerin, on the other hand, exerts pro-inflammatory effects and recruits macrophages and dendritic cells [3, 4]. Beyond their direct atherogenic action, both adipokines may notify or promote vascular damage indirectly through their associations with various parameters of lipoprotein metabolism. Thus, high adiponectin concentrations are associated with a more favorable lipid profile, including high high-density lipoprotein cholesterol (HDL-C) and low triglyceride concentrations [5, 6], while higher chemerin concentrations are associated with an unfavorable lipid profile, including greater triglycerides, high low-density lipoprotein cholesterol (LDL-C), high total cholesterol (TC), and low HDL-C concentrations [7, 8].

Lipids are transported within lipoprotein particles that vary in size, density, chemical composition, and atherosclerotic risk. Small, dense LDL particles are more atherogenic than larger, less dense LDL particles [9], while small, dense HDL particles are less protective against ASCVD compared to larger, less dense HDL particles [10]. Beyond LDL and HDL particles, other lipoprotein particles such as very low-density lipoprotein (VLDL), as well as the specific lipid composition within the particles, i.e., the cholesterol, triglyceride, and apolipoprotein (APO) content, contribute substantially to cardiovascular risk [11].

So far, only very few studies have investigated the associations between adipokines and lipoprotein subclasses [12–22]. Previous studies found that high adiponectin concentrations are positively associated with larger HDL and LDL subclasses and inversely associated with smaller VLDL subclasses [13–22]. In contrast, high chemerin concentrations were positively associated with smaller LDL and HDL subclasses and inversely with larger HDL subclasses [12]. The generalizability of the results from the existing studies is, however, restrained by limitations in their comparability due to very small sample sizes, selected populations and different measurement techniques, e.g., nuclear magnetic resonance (^1^H-NMR) spectroscopy, high-performance liquid chromatography (HPLC), and gradient gel electrophoresis.

We here hypothesize that adiponectin and chemerin are associated with lipoprotein subclasses in adults from the general population. In detail, we assume that higher adiponectin concentrations are related to a favorable lipoprotein subclass profile, while higher chemerin concentrations are related to an unfavorable profile.

## Subjects and methods

### Study population

The Study of Health in Pomerania (SHIP) is a population-based project conducted in West Pomerania, a rural region in the northeast of Germany [23]. The present work analyzed data from the SHIP-TREND cohort. This cohort is based on a stratified random sample of 8826 persons, aged 20–79 years, which was drawn from the central population registry of the German Federal State of Mecklenburg-West Pomerania. Baseline examinations were conducted between 2008 and 2012. 4420 of the invited adults chose to participate (50.1% response). The study followed the principles of the Declaration of Helsinki and was approved by the ethics committee of the University of Greifswald. All SHIP-TREND participants provided written informed consent.

**Fig. 1:**
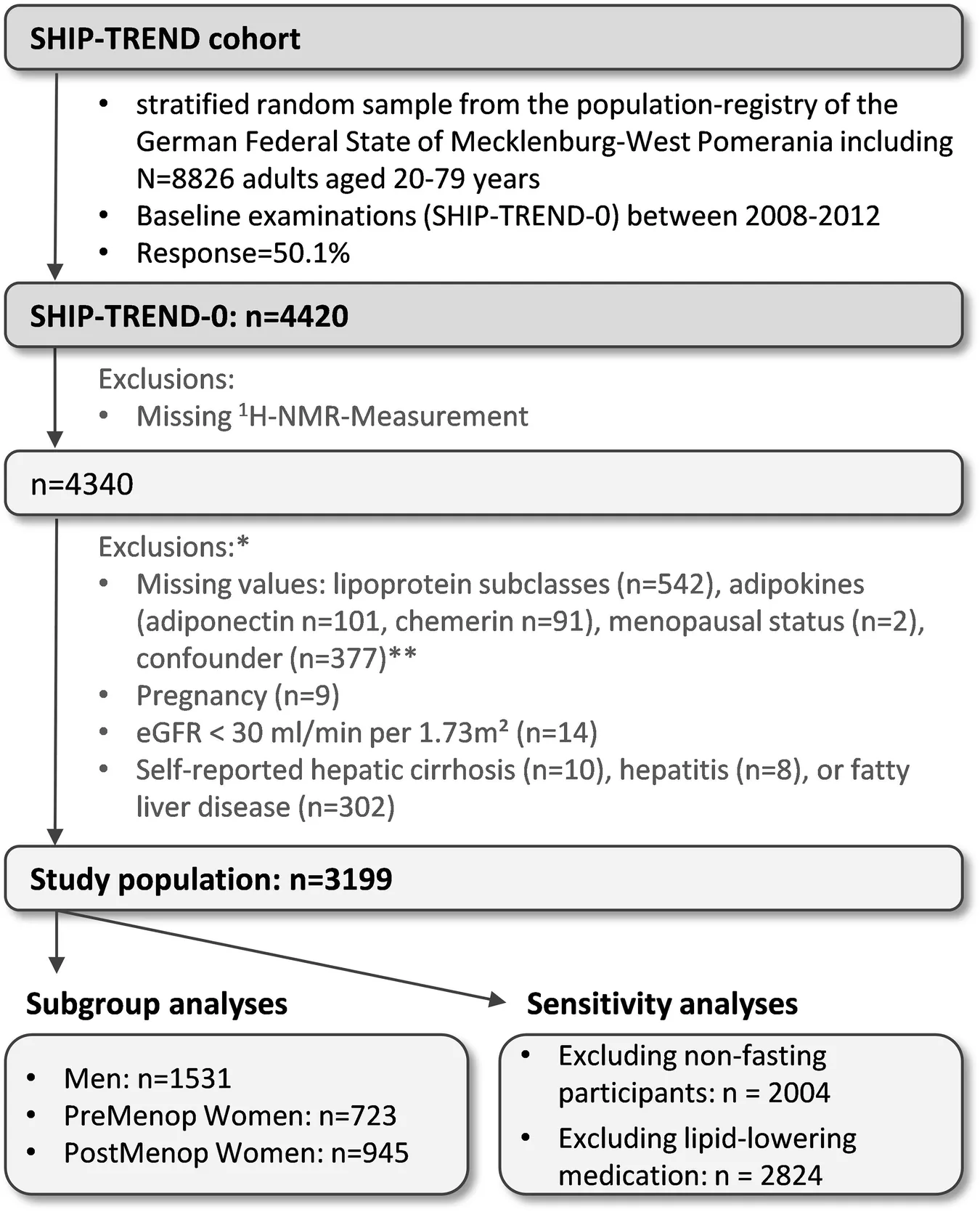
Flowchart presenting the selection of the study population. *Participants may have more than one exclusion criteria. **Considered confounders: age, sex, smoking status, waist circumference, body fat, hsCRP, glycated hemoglobin (HbA1c), eGFR, device of ^1^H-NMR measurement, use of lipid-lowering medication, fasting time. ^1^H-NMR nuclear magnetic resonance spectroscopy, eGFR estimated glomerular filtration rate, PreMenop premenopausal, PostMenop postmenopausal.

Of 4420 SHIP-TREND participants, lipoprotein subclasses were determined by ^1^H-NMR spectroscopy in 4340 individuals. Of those, 1141 participants were excluded due to the presence of at least one of the following conditions: missing values for lipoprotein subclasses, adiponectin, chemerin, menopausal status, or confounder; pregnant women; estimated glomerular filtration rate (eGFR) < 30 ml/min/1.73 m²; the presence of self-reported hepatic cirrhosis, hepatitis, or fatty liver disease. The final study population consisted of 3199 participants aged 20–83 years. It comprised 1531 men, 723 premenopausal and 945 postmenopausal women (Fig. 1).

### Interview and physical examination

Data on age, sex, sociodemographic characteristics, lifestyle, and medical histories were obtained by standardized computer-assisted personal interviews. Details are given in the supplementary information. All women younger than 40 years and women aged 40–60 years, who reported menstrual cycling, were defined as premenopausal, all remaining women as postmenopausal. Standardized measurements of body height, weight and waist circumference were performed with calibrated scales. Body fat, in kg, was measured by body impedance analysis using a Nutriguard M device and NutriPlus software (Data Input GmbH, Darmstadt, Germany). All medications that the study participants had taken in the week prior to the study interview were assigned to their active substances based on the Anatomical Therapeutic Chemical (ATC) classification system. In this way, participants could be identified who had taken lipid-lowering medication (C10).

### Laboratory methods

Blood samples were collected between 7 a.m. and 1 p.m. from the cubital vein in the supine position. The majority (62.4%, *n* = 2051) of the participants were fasting for at least 8 h at the time of blood sampling. The Chronic Kidney Disease Epidemiology Collaboration (CKD-EPI) equation [24] was used to calculate the eGFR based on cystatin C concentrations. CKD was defined as an eGFR <60 ml/min/1.73 m². Serum adiponectin and plasma chemerin concentrations were measured using commercially available enzyme-linked immunosorbent assays (Mediagnost, Reutlingen, Germany). The inter-assay coefficients of variation were 6.8% and 6.2% (adiponectin) and 5.8% and 5.5% (chemerin) for low and high concentrations, respectively.

### ^1^H-NMR-quantification of lipoprotein subclasses

A detailed description is given in the supplementary information. In short, spectra were recorded on one of three Bruker AVANCE-II 600 NMR spectrometers operated by TOPSPIN 3.2 software, equipped with a 5-mm z-gradient probe and an automated tuning and matching (ATMA) unit (Bruker Biospin, Rheinstetten, Germany). In cooperation with the Institute of Clinical Chemistry and Laboratory Medicine at the University Medicine Greifswald, Bruker developed an automatic analysis tool to quantify lipoprotein subclasses from ^1^H-NMR spectra. As part of this process, lipoprotein measurements were performed by ultracentrifugation, the gold standard method for analyzing lipoprotein subclasses [25]. Based on this development, it is now possible to quantify lipoprotein subclasses from ^1^H-NMR spectra without prior ultracentrifugation. The spectra obtained in SHIP-TREND-0 were submitted to data analysis for lipoprotein subclass and apolipoprotein analysis B.I.LISA^TM^ (Bruker BioSpin GmbH, Germany). The ^1^H-NMR approach provides results regarding the content of cholesterol, triglycerides, phospholipids, Apo-A1, Apo-A2, and Apo-B100. The content of these lipids was measured in total as well as in VLDL, intermediate-density lipoprotein (IDL), LDL, and HDL subclasses, separately.

### Statistical analyses

Characteristics of the study participants, including demographic, lifestyle, and medical data, as well as all lipoprotein subclasses were reported as median (1st quartile; 3rd quartile) for continuous data or as percentage for nominal data. Differences between men, pre- and postmenopausal women were assessed using Kruskal–Wallis or Chi-Squared tests. In the presence of statistically significant overall group differences, pairwise comparisons were performed using Wilcoxon rank-sum or Chi-Squared tests. All p-values were corrected for multiple testing using the false discovery rate (FDR), and an FDR < 0.05 was considered statistically significant.

Pearson correlation analyses were used to examine the correlations between adiponectin or chemerin and three anthropometric measures (waist circumference, BMI, body fat mass) as well as between the ^1^H-NMR variables. The latter analysis showed a strong correlation between cholesterol and phospholipid content across all examined particles (Supplementary Fig. S1). Therefore, phospholipids were not included in the main presentation of results. The corresponding results are provided in Supplementary Fig. S3.

Linear regression models were used to determine the associations of adiponectin and chemerin (exposure) with lipoprotein subclasses (outcomes). Lipoprotein data were log_2_-transformed to achieve a normal distribution. To facilitate comparisons among associations of the adipokines and lipoprotein subclasses, adiponectin, chemerin, and lipoprotein concentrations were scaled according to their standard deviations (z-transformed). All regression models were adjusted sex, age, smoking status, device of ^1^H-NMR measurement, use of lipid-lowering medication, fasting time, hsCRP, HbA1c, and eGFR. The models were further adjusted for waist circumference as a measure of fat distribution, and body fat as a measure of overall adiposity.

Sex differences and age- or menopause-specific changes in lipoprotein metabolism are well known [26, 27]. Significant interactions (*p* < 0.05) with sex or menopausal status (in women only) were detected in 73 and 101 out of 158 models (79 lipoprotein variables × 2 adipokines), respectively. Therefore, the analyses were performed in the whole study population, as well as in subgroups of men, pre- and postmenopausal women. In two sensitivity analyses the stability of the results with respect to fasting time and intake of lipid-lowering medication was examined. For this, the regression models were recalculated after exclusion of non-fasting participants or participants taking lipid-lowering medication (Fig. 1) and without the respective adjustment. Statistical significance was assumed at an FDR-value < 0.05.

All statistical analyses were performed using SAS 9.4 (SAS Institute Inc., Cary, North Carolina, USA). The code used in this study is available from the corresponding author upon request.

## Results

The general characteristics of the study population and the subpopulations are displayed in Table 1. The sex-ratio in the study population was nearly balanced (52.1% women) and more than half of women (56.7%) were postmenopausal. Men and postmenopausal women had a less favorable metabolic profile than premenopausal women, with significantly higher HbA1c, higher TC and LDL-C concentrations and higher triglycerides. This was also reflected in significantly higher proportions of metabolic and cardiovascular diseases. The medians (1st quartile; 3rd quartile) for all lipoprotein subclasses are listed in Supplementary Table S1. Statistically significant group differences were observed across nearly all lipoprotein subclasses.

**Table 1:** General characteristics of the whole study population and separated by sex and menopausal status.

Characteristics	Main analysis	Subgroup analyses			FDR < 0.05 for pairwise comparison
	Study population (*n* = 3199)	Men (*n* = 1531)	Premenopausal Women (*n* = 723)	Postmenopausal Women (*n* = 945)	
Age (years)	52.0 (40.0–64.0)	53.0 (40.0–65.0)	38.0 (31.0–44.0)	62.0 (56.0–69.0)	A, B, C
Sex (% female)	52.1	-	-	-	-
Fasting time (hours)	11.3 (2.7–13.4)	11.0 (2.5–13.1)	11.4 (3.2–13.3)	11.9 (2.8–13.9)	A, B
Smoking (%)					A, B, C
never smokers	36.6	26.1	33.3	56.2	
former smokers	35.9	44.9	28.1	27.4	
current smokers	27.5	29	38.6	16.4	
BMI (kg/m²)	27.5 (24.6–30.8)	28.0 (25.4–30.8)	25.1 (22.2–29.0)	28.0 (25.1–31.8)	A, C
Waist circumference (cm)	90.2 (80.5–100.0)	96.2 (88.5–104.6)	78.2 (71.8–88.0)	87.7 (79.8–96.7)	A, B, C
Body fat, kg	22.4 (17.6–29.1)	20.6 (16.2–25.8)	22.3 (17.1–30.5)	26.3 (20.4–33.2)	A, B, C
HbA1c (%)	5.20 (4.90–5.60)	5.30 (5.00–5.70)	4.90 (4.60–5.20)	5.40 (5.10–5.80)	A, B, C
Diabetes mellitus (%)	10.5	11.8	4.43	13.1	A, C
Arterial hypertension (%)^a^	47.1	54.4	15.2	59.5	A, B, C
Myocardial infarction (%)^a^	2.22	3.40	0.00	2.01	A, B, C
Stroke (%)^a^	1.88	2.22	0.00	2.75	A, C
hsCRP (mg/l)	1.29 (0.68–2.86)	1.12 (0.61–2.37)	1.34 (0.67–3.41)	1.54 (0.80–3.26)	A, B
Total cholesterol^b^ (mmol/l)	5.68 (4.90–6.55)	5.50 (4.72–6.40)	5.35 (4.72–6.09)	6.19 (5.46–6.99)	A, B, C
LDL-cholesterol^b^ (mmol/l)	3.44 (2.85–4.10)	3.38 (2.81–4.04)	3.19 (2.67–3.73)	3.76 (3.13–4.44)	A, B, C
HDL-cholesterol^b^ (mmol/l)	1.50 (1.28–1.78)	1.36 (1.19–1.57)	1.65 (1.41–1.91)	1.68 (1.43–1.94)	A, B
Total triglycerides^b^ (mmol/l)	1.30 (0.96–1.80)	1.41 (1.02–1.95)	1.05 (0.81–1.44)	1.35 (1.03–1.77)	A, C
Dyslipidemia (%)^a^	22.4	24.0	5.39	33.0	A, B, C
Lipid-lowering MED (% yes)	11.7	13.9	0.67	16.6	A, C
eGFR (ml/min per 1.73 m²)	111.4 (99.2– 121.0)	113.6 (101.2– 122.9)	119.5 (114.1– 125.4)	99.9 (89.0–108.0)	A, B, C
CKD (%)	2.56	2.55	0.00	4.55	A, B, C
Chemerin (ng/ml)	98.2 (83.0–17.1)	94.6 (80.5–112.2)	92.6 (78.5–110.1)	109.6 (93.5–128.8)	B, C
Adiponectin (µg/ml)	7.00 (4.68–10.20)	5.40 (3.81–7.68)	8.08 (5.66–11.00)	9.35 (6.78–13.30)	A, B, C

Continuous data are presented as median (1st quartile; 3rd quartile); nominal data are given as percentages. Overall group comparisons were performed using Kruskal-Wallis (continuous data) or Chi-Squared (categorical data) tests. *P*-values from these tests were corrected for multiple testing using the false discovery rate (FDR). All FDR values were <0.001. Pairwise comparisons for (A) men vs. premenopausal women, (B) men vs. postmenopausal women and (C) pre- vs. postmenopausal women were performed using Wilcoxon (continuous data) or Chi-Squared (categorical data) tests. P-values from these tests were corrected for multiple testing using the FDR. In case statistically significant group differences (FDR < 0.05) were observed, the respective letter (A, B, C) is printed in the table.

Diabetes mellitus was defined as self-reported physician’s diagnosis, intake of antidiabetic medication (ATC A10), a random glucose concentration ≥11.1 mmol/l or a HbA1c ≥ 6.5%. Arterial hypertension was defined as self-reported physician’s diagnosis, intake of self-reported antihypertensive medication, a systolic blood pressure ≥140 mmHg or a diastolic blood pressure ≥ 80 mmHg. Previous myocardial infarction and stroke were defined according to self-reported physician’s diagnoses. Dyslipidemia was defined as self-reported physician’s diagnosis or intake of lipid-lowering medication. Chronic kidney disease (CKD) was defined as an eGFR <60 ml/min/1.73 m^2^.

*hsCRP* high-sensitivity C-reactive protein, *LDL* low-density lipoprotein, *HDL* high-density lipoprotein, *MED* medication, *eGFR* estimated glomerular filtration rate.

^a^For *n* = 4 participants information on arterial hypertension was unavailable. For *n* = 3 participants information on myocardial infarction or stroke was unavailable. For *n* = 73 participants information on dyslipidemia was unavailable.

^b^Lipoproteins were measured using nuclear magnetic resonance spectroscopy.

Adiponectin and chemerin were significantly correlated with waist circumference, BMI and body fat mass (Supplementary Fig. S2). The correlations of adiponectin with the anthropometric markers were inverse with Pearson correlation coefficients ranging from −0.09 to −0.32, indicating weak to moderate correlations. The correlations of chemerin with the anthropometric markers were positive with Pearson correlation coefficients ranging from 0.34 to 0.43, indicating moderate correlations.

### Associations between adiponectin and ^1^H-NMR-based lipoprotein subclasses

**Fig. 2:**
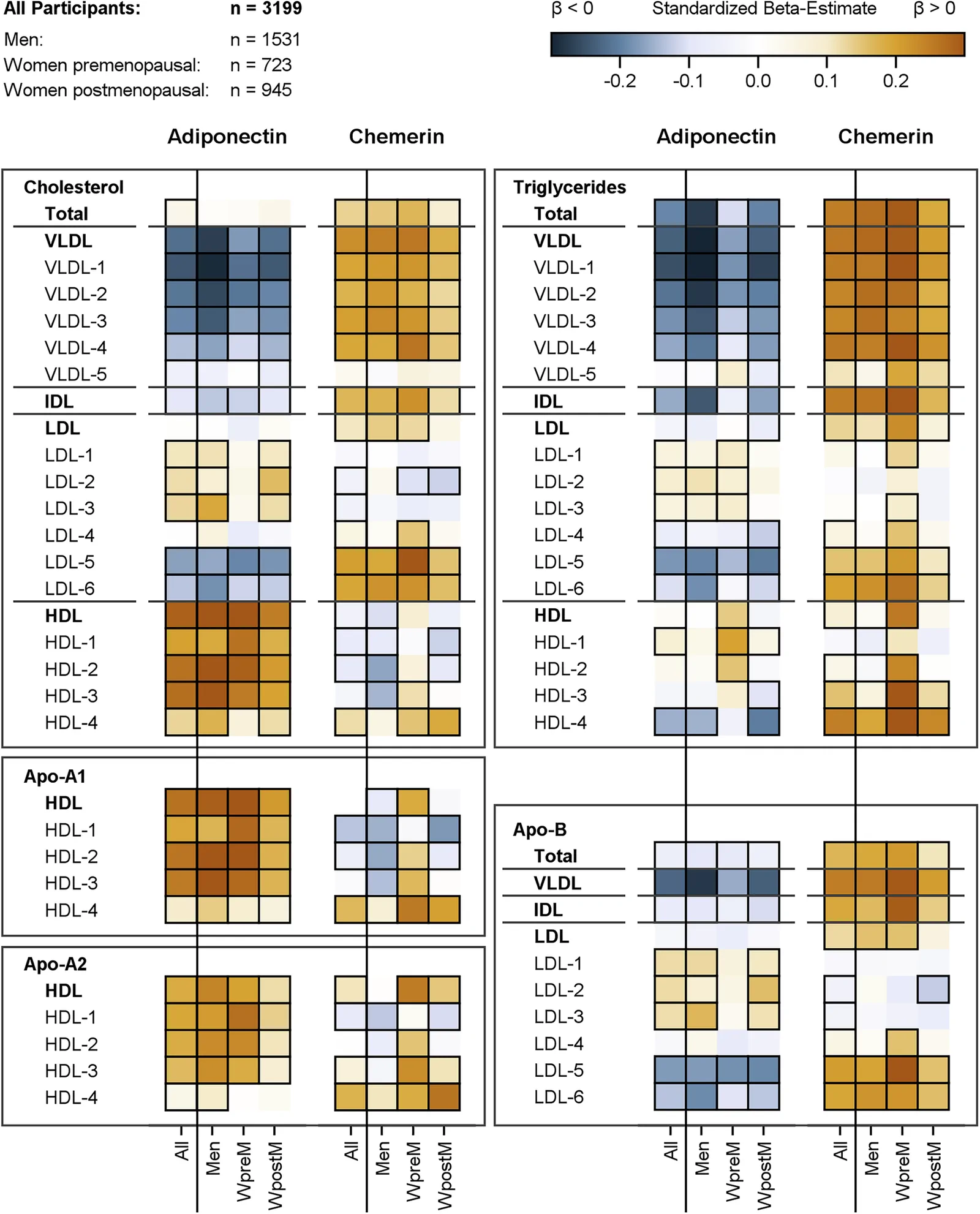
Heatmap illustrating the results from linear regression analyses for the associations of adiponectin and chemerin with ^1^H-NMR-quantified lipoprotein subclasses for the whole study population as well as for subpopulations of men, pre- and postmenopausal women. Color-coded standardized beta (ß)-estimates from linear regression analyses adjusted for age, smoking status, waist circumference, body fat, device of ^1^H-NMR measurement, use of lipid-lowering medication, fasting time, hsCRP, HbA1c, and eGFR. The model, including all participants was additionally adjusted for sex. Blue shading illustrates inverse associations (*β* < 0), orange shading illustrates positive associations (*β* > 0). Effect estimates (ß) were truncated at 0.30 for illustration. Significant associations (false discovery rate (FDR) < 0.05) are outlined. Lipoprotein concentrations were log2-transformed. Adipokines and lipoprotein concentrations were scaled according to their standard deviations (z-transformed). VLDL very low-density lipoprotein, IDL intermediate-density lipoprotein, LDL low-density lipoprotein, HDL high-density lipoprotein, Apo apolipoprotein, WpreM premenopausal women, WpostM postmenopausal women.

The effect estimates obtained from the regression models for the cholesterol, triglyceride, Apo-A1, Apo-A2 and ApoB content (Fig. 2) as well as for the phospholipid content (Supplementary Fig. S3) are illustrated in heatmaps. In these figures, blue color indicates inverse and orange color indicates positive effect estimates, and those outlined in black are statistically significant. In the whole study population as well as in the subpopulations, several clear patterns were identified. Adiponectin was significantly inversely associated with the content of cholesterol, triglycerides, and Apo-B100 in VLDL (except from VLDL-5), IDL, and small and dense LDL-5/6 particles. In contrast, significantly positive associations between adiponectin and cholesterol, triglyceride, and Apo-B100 content were detected in larger LDL-1/2/3 particles. These associations were partly absent in pre- or postmenopausal women, probably due to the reduction in sample size. Significantly positive associations in the whole study population and the three subpopulations were noted between adiponectin and the cholesterol, Apo-A1, and Apo-A2 content in HDL-particles (overall and HDL-1/2/3). In HDL-4 particles, the direction of the association was similar, but effect estimates were smaller and the statistical significance was partly missed. Finally, there was no consistent pattern in the association between adiponectin and the content of triglycerides across HDL subclasses.

### Associations between chemerin and ^1^H-NMR-based lipoprotein subclasses

When comparing the regression results obtained for adiponectin and chemerin, it became apparent that the associations were mainly in opposite directions (Fig. 2 and Supplementary Fig. S3). Thus, chemerin was significantly positively associated with the content of cholesterol, triglycerides and Apo-B100 in VLDL and IDL particles (total and subclasses, except from VLDL-5) in the whole study population and in all subpopulations. Chemerin was further significantly positively associated with the total content of cholesterol, triglycerides and Apo-B100 in LDL particles and in LDL subclasses with higher density (LDL-5/6). In contrast, in LDL-1/2/3/4 there was no clear pattern of association between chemerin and the cholesterol, triglyceride, or Apo-B100 content.

In HDL particles, clear positive associations of chemerin with the cholesterol, triglyceride, Apo-A1, and Apo-A2 content were observed in small and dense particles (HDL-4) in the whole study population and nearly all subpopulations. In HDL particles with lower density (HDL-1/2) opposite, i.e., inverse, associations were observed between chemerin and the cholesterol, Apo-A1, and Apo-A2 (HDL-1 only) content. Interestingly, these associations were generally restricted to men and postmenopausal women, while associations in premenopausal women were absent or even positive. Thus, chemerin was positively associated with Apo-A1, Apo-A2, and triglyceride content in total HDL and HDL-2/3/4 particles in premenopausal women.

Across lipoprotein subclasses, phospholipid concentrations exhibited association patterns that were highly similar to those observed for cholesterol. This pattern was consistent across analyses of both adipokines, adiponectin and chemerin (Supplementary Fig. S3).

### Regression results in the sensitivity analyses

The characteristics of the participants included in the sensitivity analyses are provided in Supplementary Table S2 and the results from the regression models in Supplementary Figs. S3 and S4. In general, the patterns of association observed in the whole study population were consistently reproduced in the sensitivity analyses, i.e., the effect direction and the statistical significance remained stable. Yet, some associations with small effect sizes in the whole study population missed statistical significance in the sensitivity analyses. The associations between adiponectin and total cholesterol content and between adiponectin and Apo-A2 content in HDL-4 particles were, for example, not replicated in the sensitivity analyses. In contrast, the associations between adiponectin and the triglyceride content in HDL-2 particles and between the Apo-B100 content in LDL particles turned significant in the two sensitivity analyses.

## Discussion

This is the first large-scale, population-based study to investigate the associations of adiponectin and chemerin with lipoprotein subfractions. The evaluation of lipoprotein subclasses allows a better differentiation of risk for ASCVD in a person than the measurement of the clinically established lipid markers LDL-C and HDL-C alone [9, 10, 28]. Small and dense LDL particles are more atherogenic than larger, less dense particles, while small and dense HDL particles are less atheroprotective than larger particles [9, 10, 28]. Also, VLDL and IDL particles, as well as their cholesterol and triglyceride content, have been associated with an increased risk of myocardial infarction and stroke [11]. The present cross-sectional study showed that a higher adiponectin concentration was associated with a more favorable lipoprotein profile, characterized by a significantly lower content of cholesterol, triglycerides, and Apo-B100 in VLDL, IDL, and small, dense LDL subclasses, as well as a significantly higher content of cholesterol and Apo-A1/A2 in HDL particles. In contrast, a high chemerin concentration was associated with an unfavorable lipoprotein profile, characterized by a significantly higher content of cholesterol, triglycerides, and Apo-B100 in VLDL, IDL, and small, dense LDL subclasses.

Phospholipid concentrations showed significant associations with both adipokines that were highly similar to those observed for cholesterol across all lipoprotein subclasses. This is expected given the strong structural and metabolic coupling of phospholipids and cholesterol within lipoprotein particles [29, 30]. Consequently, phospholipids likely reflect the same underlying variation in lipoprotein particle concentration and composition, rather than independent biological effects. Given the well-established clinical relevance of cholesterol-related measures in cardiometabolic disease, our interpretation was focused on cholesterol and triglyceride metrics.

### Associations between adiponectin and lipoprotein subclasses

Several studies have analyzed the relation between adiponectin and lipoprotein subclasses using different methods for the measurement and classification of lipoprotein subclasses (Table 2) [13–22]. These studies have consistently shown that adiponectin is inversely associated with VLDL particle size [13, 18] and the content of cholesterol and triglycerides in different VLDL subclasses [16, 22]. Similar observations were made in our study. Yet, the effect estimates for the associations across the VLDL subclasses were comparable, indicating that a respective separation is irrelevant.

**Table 2:** Overview of existing examinations analyzing the associations between adiponectin/chemerin and lipoprotein subclasses.

Examination	Population	*N*	Classification of subclasses according to	Method	Observed subclasses			Particle size			Content in subclasses	
					VLDL	LDL	HDL	VLDL	LDL	HDL	Chol	Trig
Adiponectin												
Lara-Castro et al.	Subjects with T2DM + subjects without T2DM	21 + 47	Particle size/diameter	NMR (LipoScience)	✓	✓	✓	✓	✓	✓		
Weiss et al.	Population-based Jerusalem Lipid Research Clinic study	884	Particle size/diameter	NMR (LipoScience)	✓	✓	✓	✓	✓	✓		
Goldberg et al.	Subjects with impaired glucose tolerance	2023	Particle size/diameter	NMR (LipoScience)	✓	✓	✓	✓	✓	✓		
Klobučar et al.	Healthy volunteers + patients with MetS	65 + 65	Particle size/diameter	NMR (Bruker)	✓	✓	✓				✓	✓
Shin et al.	Random subset of Cholesterol and Pharmacogenics (CAP) study	293	Particle size/diameter, mobility	GGE (Bench)		✓	✓		✓^a^		✓	
Coimbra et al.	End stage renal disease patients under chronic dialysis	194	Particle size/diameter, mobility	GGE (LipoPrint)			✓		✓		✓	
Dias et al.	T2DM patients	174	Particle size/diameter, mobility	GGE (LipoPrint)			✓				✓	
Tsubakio-Yamamoto et al.	Men who received an annual health checkup	245	Particle size/diameter	HPLC	✓	✓	✓	✓	✓	✓	✓	✓
Miyazaki et al.	(Healthy) males with MetS + males without MetS	63 + 132	Particle size/diameter	HPLC	✓	✓	✓		✓	✓	✓	✓
Calderon et al.	Subjects with T1DM + subjects without T1DM	69 + 43	Density	Ultracentrifugation			✓				✓	✓
Chemerin												
Lőrincz et al.	Non-diabetic patients with obesity + lean, healthy controls	50 + 38	Particle size/diameter, mobility	GGE (LipoPrint)		✓	✓		✓	✓	✓	

Lara-Castro et al. [14]; Weiss et al. [13]; Goldberg et al. [18]; Klobučar et al. [19]; Shin et al. [15]; Coimbra et al. [17]; Dias et al. [21]; Tsubakio-Yamamoto et al. [16]; Miyazaki et al. [22]; Calderon et al. [20]; Lőrincz et al. [12].

*T2DM* type 2 diabetes mellitus, *MetS* metabolic syndrome, *T1DM* type 1 diabetes mellitus, *NMR* nuclear magnetic resonance spectroscopy, *GGE* gradient gel electrophoresis, *HPLC* high-performance liquid chromatography, *VLDL* very low density lipoprotein, *LDL* low density lipoprotein, *HDL* high density lipoprotein, *Chol* cholesterol, *Trig* triglycerides.

^a^The peak LDL particle diameter was determined.

We observed that the associations between adiponectin and the content of cholesterol and triglycerides reversed with increasing LDL subclass density. Positive associations were detected with large, less dense LDL subclasses, while inverse associations were found with small, dense LDL subclasses. Tsubakio-Yamamoto et al. [16] detected a similar pattern between adiponectin and the cholesterol content, but inverse associations with the triglyceride content in all LDL subclasses. Both LDL-C and LDL triglycerides are linked to the initiation and progression of atherosclerosis [28], but specifically, small and dense LDL subclasses are associated with ASCVD [31, 32]. They easily enter the subendothelial space, bind to glycosaminoglycans, and are taken up by macrophages, promoting plaque formation [28].

In contrast to previous studies [15, 17, 21], we found no differences in the direction of the associations between adiponectin and the content of cholesterol in different HDL subclasses. Instead, our results clearly showed that a high adiponectin concentration was significantly associated with a high cholesterol content in all HDL subclasses. Regarding the associations between adiponectin and the triglyceride content in HDL subclasses, the present analyses did not reveal a consistent pattern. Initial examinations suggest that HDL triglycerides, unlike HDL cholesterol, are positively associated with components of the metabolic syndrome and the risk of cardiovascular diseases [11, 33]. This could explain the inverse association of adiponectin with the triglyceride content in the HDL-4 subclass.

Apo-A1/A2, present only in HDL particles, were significantly positively associated with adiponectin in all HDL subclasses. Apo-A1 is crucial for binding excess cholesterol from peripheral tissues, which is then transported to the liver for excretion as part of the reverse cholesterol transport [34]. Apo-A2 prevents further lipid accumulation on HDL and stabilizes the particles to a medium size [34]. The direction of the associations between adiponectin and Apo-B100 reversed with increasing density of LDL subclasses in the present analyses. Since each LDL and VLDL particle contains exactly one Apo-B100 molecule [28], these associations can be interpreted as reflecting the number of particles in each subclass. In line with our results, Klobučar et al. [19] have shown positive correlations between adiponectin and the content of Apo-A1/A2 in large, less-dense HDL subclasses, as well as inverse correlations with the content of Apo-B in LDL-4/5 subclasses of healthy participants.

### Associations between chemerin and lipoprotein subclasses

With a few exceptions, the observed associations between chemerin and the lipid content in the different lipoprotein subclasses were in opposite direction to those of adiponectin. This was hypothesized, as chemerin primarily exhibits pro-inflammatory functions [3].

Chemerin was significantly positively associated with the cholesterol and triglyceride content across the VLDL subclasses and the cholesterol, triglyceride, and Apo-B100 content in small, dense LDL subclasses, while findings for larger, less dense LDL subclasses were inconsistent or absent. Thus, the association of chemerin depends on the size and density of the LDL subclasses and chemerin is related to a pro-atherosclerotic LDL lipoprotein pattern. One previous study has investigated the associations between chemerin and lipoprotein subclasses [12]. In this examination, relations between chemerin and cholesterol concentrations in LDL and HDL subclasses were analyzed in 50 patients with obesity but without diabetes and 38 healthy, normal-weight controls. Similar to our results, high chemerin was associated with a high cholesterol content in small and dense LDL and HDL subclasses [12].

Chemerin associates positively with small, dense HDL subclasses in our data. Given the pro-inflammatory properties of chemerin and the observation that small, dense HDL particles are typically positively associated with cardiovascular risk [10], these findings appear plausible. In line with this, chemerin was significantly inversely related with larger, less dense HDL particles (cholesterol, Apo-A1/A2) in men and postmenopausal women. Associations in premenopausal women were absent or positive. Estrogen increases HDL and Apo-A1 production and reduces hepatic lipase activity, thereby promoting reverse cholesterol transport [35]. Moreover, higher estradiol levels have been positively associated with cholesterol efflux capacity in premenopausal, but not postmenopausal women [36]. Thus, one may speculate that in premenopausal women with high chemerin levels, estrogen counteracts part of the adverse effects on HDL metabolism, thereby preserving HDL functionality. In line with this, a previous study reported increasing HDL dysfunction during menopausal transition [26]. Yet, given the pragmatic definition of menopausal status used in our study and the lack of functional HDL data, firm conclusions cannot be drawn.

### Functions of adipokines in lipid metabolism

Experimental studies suggest that adiponectin may influence lipoprotein lipase activity [37], which is involved in the catabolism of VLDL triglycerides [38]. Furthermore, high lipoprotein lipase activity is associated with the production of larger HDL particles, which are considered to have anti-atherogenic and anti-inflammatory functions [39]. Despite the increase in lipoprotein lipase activity, no significant increase in plasma free fatty acids has been detected under the influence of adiponectin, suggesting a shift toward increased intracellular lipid catabolism [40]. Adiponectin lowers the intracellular lipid content through two primary mechanisms: First, it lowers plasma glucose levels by reducing hepatic glucose production, which in turn decreases pancreatic insulin secretion and reduces insulin-mediated lipogenesis [41]. Second, adiponectin promotes fatty acid breakdown in skeletal muscle cells by stimulating AMP-activated protein kinase, an enzyme that enhances lipid oxidation and energy expenditure [42]. This leads to increased fatty acid oxidation and a reduction in triglyceride accumulation.

The pathophysiological mechanisms to explain the relation between chemerin and lipid metabolism involve lipid mobilization, adipocyte function, and inflammation. Chemerin is known to reduce insulin sensitivity in adipose tissue [43], leading to altered lipid storage and breakdown in adipose tissue. Furthermore, it has been shown to promote adipogenesis and the differentiation of pre-adipocytes into mature adipocytes [44], which is typically associated with an increase in lipid storage [45]. In this way, chemerin indirectly influences the balance between lipid storage and mobilization in adipose tissue through changes in insulin sensitivity and inflammatory signaling [46]. In addition, chemerin promotes inflammatory processes by acting as a chemoattractant for immune cells [4], which contribute to a pro-inflammatory environment and lead to insulin resistance [47]. This insulin resistance can disrupt normal lipid metabolism, contributing to impaired lipoprotein lipase activity, fatty acid oxidation, and excess lipid storage in non-adipose tissues like the liver and muscle [48].

In general, adipokine concentrations are closely linked to the amount and the functionality of adipose tissue and may therefore represent a link between obesity and ASCVD. Other mechanisms linking these two conditions are, for example, insulin resistance, inflammation as well as an impaired renal function. To reduce the impact of these factors, we adjusted our analyses for Hba1C, eGFR, hsCRP, waist circumference, and body fat, and our findings suggest that the identified associations are largely independent of these conditions. Moreover, we examined the stability of our results after exclusion of non-fasting individuals and those taking lipid-lowering medication. While circulating adiponectin [49] or chemerin concentrations [50] are not affected by fasting status as long as daily energy intake is not drastically reduced, circulating lipid concentrations, especially triglycerides, are altered postprandially [51]. Also, effects of statins and fibrates, the most commonly used lipid-lowering agents, on adipokine concentrations [52–54] have been reported. Both increase adiponectin levels [52, 53], while statins additionally decrease chemerin concentrations [54]. Of the 134 statistically significant associations identified in our main analyses, 130 and 131 remained significant after excluding non-fasting participants and those on lipid-lowering medication, respectively. In both sensitivity analyses, four additional associations reached statistical significance, but there was no consistent pattern in the gain or loss of associations. We therefore concluded that neither condition had a meaningful impact on the results.

### Strengths and limitations

SHIP examinations are characterized by highly standardized procedures, ensuring high data quality. The use of the ^1^H-NMR method to study the lipoprotein profile provided an exceptionally wide view on lipid metabolism. Potential major limitations to the observed associations and the generalizability of our findings result from the cross-sectional design of the present study. It prohibits drawing conclusions about causality and prevents differentiation between cause and effect in the observed associations. In our study population, about a quarter of individuals had normal weight, two quarters had overweight and one quarter had obesity, which suggests that metabolic alterations with respect to glucose and lipid metabolism are common. Although our findings were independent of several related covariates, including lipid-lowering medication, hsCRP, HbA1c, waist circumference, and body fat mass, residual confounding related to metabolic dysregulation cannot be excluded. Finally, whether the observed effects on lipoprotein metabolism translate into actual atherosclerosis promotion in our participants cannot be determined from our study. While ^1^H-NMR spectroscopy is a robust and reproducible method requiring minimal sample preparation and data processing time, its high costs currently limit its widespread implementation in routine clinical practice.

### Conclusion

The highly consistent results presented here demonstrate that high adiponectin concentrations are associated with a favorable, anti-atherogenic lipoprotein profile, whereas the opposite pattern is observed for chemerin. Experimental and longitudinal research is needed to clarify causality and the precise mechanisms involved in this context.

## Supplementary information

Supplemental Material

## Data Availability

Restrictions apply to the availability of data generated or analyzed during this study to preserve patient confidentiality or because they were used under license. Data can be applied for following a standardized procedure: http://www2.medizin.uni-greifswald.de/cm/fv/ship/daten-beantragen/.
