# Supplementary material for: Linking circulating chemerin and adiponectin to lipoprotein subclasses measured by 1H-NMR spectroscopy in 3199 participants from the study of health in Pomerania
Source: Int J Obes (Lond). 2026 Jun 26;50(9):1978–87. doi: 10.1038/s41366-026-02138-8 (PMC13649557; doi:10.1038/s41366-026-02138-8)
Supplement: Supplementary file 1 [file 41366_2026_2138_MOESM1_ESM.docx]

**Linking Circulating Chemerin and Adiponectin to Lipoprotein Subclasses Measured by ^1^H-NMR spectroscopy in 3199 Participants from the Study of Health in Pomerania**

Benjamin Spindler^1^, Anke Hannemann^1,2^, Henry Völzke^3^, Stefan Groß^2,4^, Martin Bahls^2,4^, Kathrin Budde^1^, Astrid Petersmann^1,5^, Ann-Kristin Henning^1^, Theresa Winter^1^, Stephanie Könemann^2,4^, Marcus Dörr^2,3^, Matthias Nauck^1,2^, Nele Friedrich^1,2^, Stephanie Zylla^1,2^

1) Institute of Clinical Chemistry and Laboratory Medicine, University Medicine Greifswald, Germany

2) DZHK (German Center for Cardiovascular Research), partner site Greifswald, Germany

3) Institute for Community Medicine, University Medicine Greifswald, Germany

4) Department of Internal Medicine B, University Medicine Greifswald, Germany

5) Institute for Clinical Chemistry and Laboratory Medicine, University Medicine Oldenburg, Oldenburg, Germany

**Supplementary Information**

**Interview and physical examination**

Previous myocardial infarction and stroke were defined according to self-reported physician’s diagnoses. Arterial hypertension was defined as self-reported physician’s diagnosis, intake of self-reported antihypertensive medication, a systolic blood pressure ≥140 mmHg or a diastolic blood pressure ≥ 80 mmHg. The participants were categorized according to their smoking status into current, former, or never smokers. Dyslipidemia was defined as self-reported physician’s diagnosis or intake of lipid-lowering medication. Diabetes mellitus was defined as self-reported physician’s diagnosis, intake of antidiabetic medication (ATC A10), a random glucose concentration ≥11.1 mmol/l or a HbA1c ≥6.5%.

Standardized measurements of body height, weight and waist circumference were performed with calibrated scales. BMI was calculated as weight (kg)/height² (m²). The participant’s waist circumference was measured to the nearest 0.1 cm using an inelastic tape midway between the lower rib margin and the iliac crest in the horizontal plane.

**^1^H-NMR-quantification of lipoprotein subclasses**

Plasma samples were stored frozen at -80°C until analysis. After thawing, 250 μL of plasma were mixed with 250 μL of phosphate buffer (prepared with D2O and contained sodium 3-trimethylsilyl-(2,2,3,3-D4)-1-propionate (TSP) as reference (pH 7.4)). Spectra were recorded on one of three Bruker AVANCE-II 600 NMR spectrometers operated by TOPSPIN 3.2 software, equipped with a 5-mm z-gradient probe and automated tuning and matching (ATMA) unit (Bruker Biospin, Rheinstetten, Germany). In cooperation with the Institute of Clinical Chemistry and Laboratory Medicine at the University Medicine Greifswald, Bruker developed an automatic analysis tool to quantify lipoprotein subclasses from NMR spectra. In the framework of this process the lipoprotein measurements were performed by ultracentrifugation, the gold standard method to analyze lipoproteins (1), in Greifswald. Specimens were automatically delivered to the spectrometer via SampleJet (Bruker Biospin, Rheinstetten, Germany) into standard 5 mm NMR tubes. The acquisition temperature was set to 310°K. A standard 1-dimensional ^1^H-NMR pulse sequence with suppression of the water peak (NOESYPREAST) was used. The sequence has the form –RD-gz,1-90°-t-90°-tm-gz,2-ACQ, where RD is the relaxation delay (4 sec), t is a short delay (~3 µsec), 90° represents the 90° RF hard pulse, tm is the mixing time (10 msec), gz,1 and gz,2 are the magnetic field z gradients both applied for 1 msec, and ACQ is the acquisition period (2.7 sec) collecting 98304 data points at a sweep width of 30 ppm. The receiver gain is set at 90.5 for all experiments. For pre-processing, a line broadening of 0.3 Hz, a zero-filling to produce 128k data points, and a first-order phase correction of 0.0 were applied. Spectral processing included zero filling, line broadening, Fourier transformation and referencing of the chemical shift, and determination of the spectral intensity per one mmol protons for quantitative referencing. Chemical shifts of plasma spectra were referenced to the CH3-group signal of alanine, adjusting it to 1.48 ppm. Spectra were segmented into N = 450 consecutive integrated spectral regions (buckets) of fixed width covering the region from 0.3 ppm to 1.4 ppm. Two sub-regions, i.e., 1.31 - 1.35 ppm and 1.16 - 1.21 ppm, were excluded from binning in order to avoid unwanted influences coming from lactate and ethanol CH3-group signals in later application of the method in typical population cohorts. Finally, the spectra were submitted to data analysis for lipoprotein subclass and apolipoprotein analysis B.I.LISA^TM^ (Bruker BioSpin GmbH Germany).

All laboratory methods were performed by skilled technical personnel and according to manufacturers’ recommendations. Based on replicates of a pooled plasma sample, which were measured during the year 2023 on two different 600 MHz NMR spectrometers, the coefficients of variation (CV) for total cholesterol, LDL-cholesterol, HDL-cholesterol, and triglycerides varied between 2.85% - 4.69%, 3.77% - 6.13%, 3.20% - 5.43% and 4.85% - 5.20%, respectively.

The ^1^H-NMR approach provides results about the content of cholesterol, triglycerides, phospholipids, apolipoprotein (Apo)-A1, Apo-A2, and Apo-B100. If possible, the content of these lipids was measured in total as well as in VLDL, intermediate-density lipoprotein (IDL), LDL, and HDL subclasses, separately. Total cholesterol, LDL-cholesterol, HDL-cholesterol, and triglycerides measured by ^1^H-NMR were compared to standard laboratory measurements (Dimension Vista 1500, Siemens Healthineers, Erlangen, Germany) using Passing-Bablok regression and Pearson correlation in 4340 SHIP-TREND participants. The agreement between the methods was good for all four measurands, with Pearson correlation coefficients between 0.90 and 0.97 (data not shown). Further, high correlation coefficients were observed between several of the ^1^H-NMR variables (Supplementary Figure S1). Specifically, the cholesterol and phospholipid content were highly correlated in all of the examined particles.

**REFERENCES:**

1.	Baumstark MW, Kreutz W, Berg A, Frey I, Keul J. Structure of human low-density lipoprotein subfractions, determined by X-ray small-angle scattering. Biochim Biophys Acta. 1990;1037(1):48-57.

**Supplementary Figures**

**Supplementary Fig. S1:** Correlation matrices showing within-particle Pearson correlations of cholesterol (Chol), phospholipid (PL) and triglyceride (TG) content in LDL subclasses (left), VLDL subclasses (middle) and HDL subclasses (right). VLDL = very low-density lipoprotein; IDL = intermediate-density lipoprotein; LDL = low-density lipoprotein; HDL = high-density lipoprotein; Apo = apolipoprotein.

**Supplementary Fig. S2:** Scatterplots illustrating the correlations between waist circumference, BMI, body fat mass and adiponectin (upper panel) or chemerin (lower panel) in the main study population (n=3199). Pearson correlation coefficients (*r*) and corresponding p-values are displayed in the upper right corner.

**Supplementary Fig. S3:** Heatmap illustrating the results from linear regression analyses for the associations of adiponectin and chemerin with the phospholipid content in ^1^H-NMR-quantified lipoprotein subclasses for the whole study population as well as for subpopulations of men, pre- and postmenopausal women. Color-coded standardized beta (ß)-estimates from linear regression analyses adjusted for age, smoking status, waist circumference, body fat, device of ^1^H-NMR measurement, use of lipid-lowering medication, fasting time, hsCRP, HbA1c, and eGFR. The model including all participants was additionally adjusted for sex. Blue shading illustrates inverse associations (β<0), orange shading illustrates positive associations (β>0). Effect estimates (ß) were truncated at 0.30 for illustration. Significant associations (false discovery rate (FDR)<0.05) are outlined. Lipoprotein concentrations were log2-transformed. Adipokines and lipoprotein concentrations were scaled according to their standard deviations (z-transformed). VLDL = very low-density lipoprotein; IDL = intermediate-density lipoprotein; LDL = low-density lipoprotein; HDL = high-density lipoprotein; Apo = apolipoprotein; WpreM = premenopausal women; WpostM = postmenopausal women

**Supplementary Fig. S4:** Heatmap illustrating the results from linear regression analyses for the associations of adiponectin and chemerin with ^1^H-NMR-quantified lipoprotein subclasses for the whole study population as well as for subpopulations excluding non-fasting participants or those taking lipid-lowering medication. Color-coded standardized beta (ß)-estimates from linear regression analyses adjusted for age, sex, smoking status, waist circumference, body fat, device of ^1^H-NMR measurement, fasting time, hsCRP, HbA1c, and eGFR. Model including all participants and fasting participants were additionally adjusted for intake of lipid-lowering medication. Blue shading illustrates inverse associations (β<0), orange shading illustrates positive associations (β>0). Effect estimates were truncated at 0.30 for illustration. Significant associations (false discovery rate (FDR)<0.05) are outlined. Lipoprotein concentrations were log2-transformed. Adipokines and lipoprotein concentrations were scaled according to their standard deviations (z-transformed). VLDL = very low-density lipoprotein; IDL = intermediate-density lipoprotein; LDL = low-density lipoprotein; HDL = high-density lipoprotein; Apo = apolipoprotein

**Supplementary Tables**

| **Lipoprotein Subclass** | **All participants (n=3199)** | **Men  (n=1531)** | **Premenop.  Women (n=723)** | **Postmenop. Women (n=945)** | **FDR, overall** | **FDR, men vs. premenop. women** | **FDR, men vs. postmenop. women** | **FDR, pre- vs. postmenop. women** |
| --- | --- | --- | --- | --- | --- | --- | --- | --- |
| **Cholesterol** |  |  |  |  |  |  |  |  |
| Total Cholesterol (mmol/l) | 5.68 (4.90-6.55) | 5.50 (4.72-6.40) | 5.35 (4.72-6.09) | 6.19 (5.46-6.99) | <0.001 | 0.009 | <0.001 | <0.001 |
| VLDL Cholesterol (mmol/l) | 0.46 (0.30-0.71) | 0.54 (0.34-0.80) | 0.34 (0.22-0.51) | 0.46 (0.30-0.71) | <0.001 | <0.001 | <0.001 | <0.001 |
| VLDL1 Cholesterol (mmol/l) | 0.13 (0.07-0.23) | 0.16 (0.09-0.27) | 0.09 (0.04-0.15) | 0.13 (0.07-0.21) | <0.001 | <0.001 | <0.001 | <0.001 |
| VLDL2 Cholesterol (mmol/l) | 0.06 (0.03-0.11) | 0.08 (0.04-0.14) | 0.04 (0.02-0.07) | 0.06 (0.03-0.10) | <0.001 | <0.001 | <0.001 | <0.001 |
| VLDL3 Cholesterol (mmol/l) | 0.09 (0.05-0.14) | 0.10 (0.06-0.16) | 0.07 (0.04-0.10) | 0.09 (0.05-0.14) | <0.001 | <0.001 | <0.001 | <0.001 |
| VLDL4 Cholesterol (mmol/l) | 0.12 (0.08-0.18) | 0.13 (0.09-0.19) | 0.10 (0.07-0.14) | 0.13 (0.09-0.19) | <0.001 | <0.001 | 0.801 | <0.001 |
| VLDL5 Cholesterol (mmol/l) | 0.03 (0.02-0.04) | 0.03 (0.02-0.04) | 0.03 (0.02-0.04) | 0.04 (0.03-0.05) | <0.001 | 0.991 | <0.001 | <0.001 |
| IDL Cholesterol (mmol/l) | 0.32 (0.22-0.46) | 0.33 (0.23-0.47) | 0.26 (0.18-0.36) | 0.37 (0.26-0.50) | <0.001 | <0.001 | <0.001 | <0.001 |
| LDL Cholesterol (mmol/l) | 3.44 (2.85-4.10) | 3.38 (2.81-4.04) | 3.19 (2.67-3.73) | 3.76 (3.13-4.44) | <0.001 | <0.001 | <0.001 | <0.001 |
| LDL1 Cholesterol (mmol/l) | 0.52 (0.41-0.67) | 0.46 (0.36-0.59) | 0.53 (0.41-0.65) | 0.63 (0.50-0.79) | <0.001 | <0.001 | <0.001 | <0.001 |
| LDL2 Cholesterol (mmol/l) | 0.46 (0.35-0.58) | 0.44 (0.33-0.55) | 0.48 (0.37-0.58) | 0.51 (0.38-0.65) | <0.001 | <0.001 | <0.001 | <0.001 |
| LDL3 Cholesterol (mmol/l) | 0.49 (0.35-0.65) | 0.44 (0.31-0.60) | 0.51 (0.39-0.63) | 0.56 (0.42-0.73) | <0.001 | <0.001 | <0.001 | <0.001 |
| LDL4 Cholesterol (mmol/l) | 0.58 (0.43-0.76) | 0.55 (0.41-0.75) | 0.57 (0.41-0.72) | 0.64 (0.49-0.81) | <0.001 | 0.906 | <0.001 | <0.001 |
| LDL5 Cholesterol (mmol/l) | 0.64 (0.49-0.82) | 0.70 (0.53-0.86) | 0.54 (0.40-0.68) | 0.63 (0.50-0.81) | <0.001 | <0.001 | <0.001 | <0.001 |
| LDL6 Cholesterol (mmol/l) | 0.64 (0.50-0.83) | 0.71 (0.55-0.91) | 0.51 (0.41-0.65) | 0.65 (0.52-0.81) | <0.001 | <0.001 | <0.001 | <0.001 |
| HDL Cholesterol (mmol/l) | 1.50 (1.28-1.78) | 1.36 (1.19-1.57) | 1.65 (1.41-1.91) | 1.68 (1.43-1.94) | <0.001 | <0.001 | <0.001 | 0.137 |
| HDL1 Cholesterol (mmol/l) | 0.41 (0.30-0.56) | 0.33 (0.26-0.43) | 0.48 (0.35-0.65) | 0.51 (0.39-0.67) | <0.001 | <0.001 | <0.001 | <0.001 |
| HDL2 Cholesterol (mmol/l) | 0.22 (0.18-0.28) | 0.19 (0.16-0.23) | 0.26 (0.21-0.32) | 0.26 (0.22-0.32) | <0.001 | <0.001 | <0.001 | 0.866 |
| HDL3 Cholesterol (mmol/l) | 0.28 (0.23-0.34) | 0.25 (0.21-0.30) | 0.32 (0.27-0.37) | 0.31 (0.27-0.36) | <0.001 | <0.001 | <0.001 | 0.022 |
| HDL4 Cholesterol (mmol/l) | 0.55 (0.48-0.63) | 0.55 (0.48-0.64) | 0.55 (0.48-0.61) | 0.56 (0.49-0.63) | 0.146 | 0.078 | 0.913 | 0.075 |
| **Triglycerides** |  |  |  |  |  |  |  |  |
| Total Triglycerides (mmol/l) | 1.30 (0.96-1.80) | 1.41 (1.02-1.95) | 1.05 (0.81-1.44) | 1.35 (1.03-1.77) | <0.001 | <0.001 | 0.069 | <0.001 |
| VLDL Triglycerides (mmol/l) | 0.85 (0.56-1.26) | 0.99 (0.64-1.42) | 0.62 (0.44-0.93) | 0.84 (0.58-1.21) | <0.001 | <0.001 | <0.001 | <0.001 |
| VLDL1 Triglycerides (mmol/l) | 0.38 (0.23-0.62) | 0.46 (0.28-0.71) | 0.26 (0.17-0.43) | 0.37 (0.24-0.58) | <0.001 | <0.001 | <0.001 | <0.001 |
| VLDL2 Triglycerides (mmol/l) | 0.15 (0.09-0.24) | 0.18 (0.11-0.28) | 0.10 (0.06-0.17) | 0.14 (0.09-0.22) | <0.001 | <0.001 | <0.001 | <0.001 |
| VLDL3 Triglycerides (mmol/l) | 0.13 (0.09-0.20) | 0.16 (0.10-0.23) | 0.10 (0.07-0.15) | 0.13 (0.09-0.20) | <0.001 | <0.001 | <0.001 | <0.001 |
| VLDL4 Triglycerides (mmol/l) | 0.12 (0.09-0.15) | 0.12 (0.09-0.16) | 0.10 (0.07-0.13) | 0.12 (0.09-0.16) | <0.001 | <0.001 | 0.935 | <0.001 |
| VLDL5 Triglycerides (mmol/l) | 0.03 (0.02-0.04) | 0.03 (0.02-0.04) | 0.03 (0.02-0.04) | 0.03 (0.03-0.04) | <0.001 | 0.775 | <0.001 | <0.001 |
| IDL Triglycerides (mmol/l) | 0.12 (0.07-0.20) | 0.14 (0.08-0.23) | 0.09 (0.05-0.15) | 0.13 (0.08-0.20) | <0.001 | <0.001 | 0.137 | <0.001 |
| LDL Triglycerides (mmol/l) | 0.25 (0.21-0.30) | 0.24 (0.20-0.29) | 0.23 (0.20-0.28) | 0.27 (0.23-0.33) | <0.001 | 0.073 | <0.001 | <0.001 |
| LDL1 Triglycerides (mmol/l) | 0.06 (0.05-0.08) | 0.05 (0.04-0.07) | 0.06 (0.05-0.08) | 0.07 (0.06-0.09) | <0.001 | <0.001 | <0.001 | <0.001 |
| LDL2 Triglycerides (mmol/l) | 0.03 (0.02-0.03) | 0.02 (0.02-0.03) | 0.03 (0.02-0.04) | 0.03 (0.03-0.04) | <0.001 | <0.001 | <0.001 | <0.001 |
| LDL3 Triglycerides (mmol/l) | 0.03 (0.02-0.04) | 0.03 (0.02-0.03) | 0.03 (0.02-0.04) | 0.03 (0.03-0.04) | <0.001 | <0.001 | <0.001 | <0.001 |
| LDL4 Triglycerides (mmol/l) | 0.03 (0.03-0.05) | 0.03 (0.02-0.04) | 0.03 (0.03-0.04) | 0.04 (0.03-0.05) | <0.001 | 0.749 | <0.001 | <0.001 |
| LDL5 Triglycerides (mmol/l) | 0.04 (0.03-0.05) | 0.04 (0.03-0.05) | 0.03 (0.02-0.04) | 0.04 (0.03-0.05) | <0.001 | <0.001 | 0.189 | <0.001 |
| LDL6 Triglycerides (mmol/l) | 0.05 (0.04-0.06) | 0.05 (0.04-0.07) | 0.04 (0.03-0.05) | 0.05 (0.04-0.06) | <0.001 | <0.001 | 0.030 | <0.001 |
| HDL Triglycerides (mmol/l) | 0.14 (0.12-0.17) | 0.13 (0.11-0.16) | 0.15 (0.12-0.19) | 0.15 (0.13-0.18) | <0.001 | <0.001 | <0.001 | 0.007 |
| HDL1 Triglycerides (mmol/l) | 0.04 (0.03-0.06) | 0.03 (0.03-0.05) | 0.05 (0.03-0.07) | 0.05 (0.04-0.07) | <0.001 | <0.001 | <0.001 | <0.001 |
| HDL2 Triglycerides (mmol/l) | 0.02 (0.02-0.03) | 0.02 (0.02-0.03) | 0.03 (0.02-0.03) | 0.03 (0.02-0.03) | <0.001 | <0.001 | <0.001 | 0.234 |
| HDL3 Triglycerides (mmol/l) | 0.03 (0.02-0.04) | 0.03 (0.02-0.03) | 0.03 (0.02-0.04) | 0.03 (0.03-0.04) | <0.001 | <0.001 | <0.001 | 0.692 |
| HDL4 Triglycerides (mmol/l) | 0.05 (0.04-0.06) | 0.05 (0.04-0.06) | 0.05 (0.04-0.06) | 0.05 (0.04-0.06) | <0.001 | <0.001 | 0.003 | 0.111 |
| **Phospholipids** |  |  |  |  |  |  |  |  |
| VLDL Phospholipids (mmol/l) | 0.24 (0.16-0.35) | 0.28 (0.19-0.38) | 0.19 (0.13-0.27) | 0.24 (0.16-0.33) | <0.001 | <0.001 | <0.001 | <0.001 |
| VLDL1 Phospholipids (mmol/l) | 0.07 (0.04-0.11) | 0.08 (0.05-0.13) | 0.05 (0.03-0.08) | 0.07 (0.04-0.10) | <0.001 | <0.001 | <0.001 | <0.001 |
| VLDL2 Phospholipids (mmol/l) | 0.04 (0.03-0.06) | 0.05 (0.03-0.07) | 0.03 (0.02-0.04) | 0.04 (0.03-0.06) | <0.001 | <0.001 | <0.001 | <0.001 |
| VLDL3 Phospholipids (mmol/l) | 0.05 (0.03-0.07) | 0.06 (0.04-0.08) | 0.04 (0.03-0.06) | 0.05 (0.03-0.07) | <0.001 | <0.001 | <0.001 | <0.001 |
| VLDL4 Phospholipids (mmol/l) | 0.06 (0.04-0.07) | 0.06 (0.05-0.08) | 0.05 (0.04-0.06) | 0.06 (0.04-0.08) | <0.001 | <0.001 | 0.430 | <0.001 |
| VLDL5 Phospholipids (mmol/l) | 0.02 (0.01-0.02) | 0.02 (0.01-0.02) | 0.02 (0.01-0.02) | 0.02 (0.01-0.03) | <0.001 | 0.004 | <0.001 | <0.001 |
| IDL Phospholipids (mmol/l) | 0.09 (0.07-0.12) | 0.09 (0.07-0.13) | 0.07 (0.05-0.10) | 0.10 (0.08-0.13) | <0.001 | <0.001 | 0.030 | <0.001 |
| LDL Phospholipids (mmol/l) | 0.83 (0.71-0.98) | 0.81 (0.68-0.95) | 0.78 (0.68-0.91) | 0.91 (0.77-1.05) | <0.001 | 0.009 | <0.001 | <0.001 |
| LDL1 Phospholipids (mmol/l) | 0.13 (0.11-0.17) | 0.12 (0.10-0.15) | 0.14 (0.11-0.17) | 0.16 (0.13-0.20) | <0.001 | <0.001 | <0.001 | <0.001 |
| LDL2 Phospholipids (mmol/l) | 0.11 (0.09-0.14) | 0.11 (0.08-0.13) | 0.12 (0.09-0.14) | 0.12 (0.10-0.16) | <0.001 | <0.001 | <0.001 | <0.001 |
| LDL3 Phospholipids (mmol/l) | 0.12 (0.09-0.15) | 0.11 (0.08-0.14) | 0.12 (0.10-0.15) | 0.14 (0.11-0.17) | <0.001 | <0.001 | <0.001 | <0.001 |
| LDL4 Phospholipids (mmol/l) | 0.14 (0.10-0.18) | 0.13 (0.10-0.17) | 0.14 (0.10-0.17) | 0.15 (0.12-0.19) | <0.001 | <0.001 | <0.001 | <0.001 |
| LDL5 Phospholipids (mmol/l) | 0.15 (0.12-0.19) | 0.16 (0.13-0.20) | 0.13 (0.10-0.16) | 0.15 (0.12-0.19) | <0.001 | <0.001 | <0.001 | <0.001 |
| LDL6 Phospholipids (mmol/l) | 0.16 (0.13-0.20) | 0.17 (0.14-0.21) | 0.13 (0.11-0.16) | 0.16 (0.13-0.19) | <0.001 | <0.001 | <0.001 | <0.001 |
| HDL Phospholipids (mmol/l) | 0.90 (0.76-1.07) | 0.79 (0.69-0.91) | 1.02 (0.89-1.18) | 1.01 (0.87-1.16) | <0.001 | <0.001 | <0.001 | 0.119 |
| HDL1 Phospholipids (mmol/l) | 0.21 (0.15-0.29) | 0.16 (0.13-0.21) | 0.26 (0.19-0.36) | 0.26 (0.20-0.36) | <0.001 | <0.001 | <0.001 | 0.429 |
| HDL2 Phospholipids (mmol/l) | 0.15 (0.12-0.19) | 0.13 (0.10-0.15) | 0.18 (0.15-0.22) | 0.18 (0.15-0.21) | <0.001 | <0.001 | <0.001 | 0.048 |
| HDL3 Phospholipids (mmol/l) | 0.19 (0.16-0.23) | 0.17 (0.14-0.20) | 0.22 (0.19-0.26) | 0.21 (0.18-0.25) | <0.001 | <0.001 | <0.001 | <0.001 |
| HDL4 Phospholipids (mmol/l) | 0.33 (0.30-0.37) | 0.33 (0.29-0.37) | 0.34 (0.31-0.38) | 0.34 (0.30-0.38) | <0.001 | <0.001 | <0.001 | 0.022 |
| **Apo-A1** |  |  |  |  |  |  |  |  |
| HDL Apo-A1 (mg/dl) | 158 (139-179) | 146 (131-162) | 170 (149-191) | 170 (152-191) | <0.001 | <0.001 | <0.001 | 0.960 |
| HDL1 ApoA1 (mg/dl) | 22.7 (16.3-33.5) | 18.4 (13.7-24.6) | 28.8 (20.5-42.1) | 28.7 (21.1-40.6) | <0.001 | <0.001 | <0.001 | 0.718 |
| HDL2 ApoA1 (mg/dl) | 19.9 (16.5-24.2) | 17.4 (14.7-20.2) | 23.1 (19.5-27.9) | 22.5 (19.1-25.9) | <0.001 | <0.001 | <0.001 | 0.002 |
| HDL3 ApoA1 (mg/dl) | 28.4 (23.4-33.7) | 24.9 (21.2-29.3) | 31.9 (27.3-37.6) | 31.2 (26.9-35.7) | <0.001 | <0.001 | <0.001 | 0.002 |
| HDL4 ApoA1 (mg/dl) | 82.3 (73.7-91.1) | 82.1 (73.3-91.5) | 81.5 (73.4-89.4) | 82.9 (74.4-91.5) | 0.077 | 0.346 | 0.125 | 0.022 |
| **Apo-A2** |  |  |  |  |  |  |  |  |
| HDL Apo-A2 (mg/dl) | 35.6 (31.9-39.7) | 34.0 (30.5-38.1) | 37.4 (33.7-41.8) | 36.9 (33.4-40.6) | <0.001 | <0.001 | <0.001 | 0.024 |
| HDL1 ApoA2 (mg/dl) | 2.6 (1.8-3.7) | 1.9 (1.4-2.7) | 3.3 (2.4-4.7) | 3.2 (2.4-4.3) | <0.001 | <0.001 | <0.001 | 0.130 |
| HDL2 ApoA2 (mg/dl) | 3.9 (3.0-4.8) | 3.3 (2.7-4.0) | 4.5 (3.7-5.4) | 4.4 (3.7-5.1) | <0.001 | <0.001 | <0.001 | 0.011 |
| HDL3 ApoA2 (mg/dl) | 7.3 (6.2-8.5) | 6.6 (5.7-7.8) | 8.0 (7.0-9.5) | 7.8 (6.9-9.0) | <0.001 | <0.001 | <0.001 | 0.005 |
| HDL4 ApoA2 (mg/dl) | 20.8 (18.2-23.5) | 21.2 (18.4-24.0) | 20.5 (18.1-22.8) | 20.5 (17.9-23.2) | <0.001 | <0.001 | <0.001 | 0.989 |
| **Apo-B** |  |  |  |  |  |  |  |  |
| Total Apo-B (mg/dl) | 98.6 (82.4-116) | 99.4 (82.6-116) | 88.8 (74.8-103) | 107 (90.0-124) | <0.001 | <0.001 | <0.001 | <0.001 |
| VLDL Apo-B (mg/dl) | 8.5 (6.2-11.6) | 9.4 (6.8-12.7) | 6.9 (5.0-9.2) | 8.6 (6.4-11.7) | <0.001 | <0.001 | <0.001 | <0.001 |
| IDL Apo-B (mg/dl) | 5.3 (4.0-7.0) | 5.3 (4.0-7.0) | 4.6 (3.5-6.2) | 6.0 (4.5-7.7) | <0.001 | <0.001 | <0.001 | <0.001 |
| LDL Apo-B (mg/dl) | 83.2 (68.7-98.9) | 82.9 (67.7-98.4) | 75.8 (63.3-88.8) | 90.6 (75.7-107) | <0.001 | <0.001 | <0.001 | <0.001 |
| LDL1 ApoB (mg/dl) | 10.5 (8.3-13.5) | 9.2 (7.3-11.8) | 10.7 (8.6-13.1) | 12.7 (10.3-15.8) | <0.001 | <0.001 | <0.001 | <0.001 |
| LDL2 ApoB (mg/dl) | 10.0 (8.0-12.4) | 9.4 (7.5-11.6) | 10.2 (8.4-12.4) | 11.0 (8.6-13.6) | <0.001 | <0.001 | <0.001 | <0.001 |
| LDL3 ApoB (mg/dl) | 11.0 (8.3-14.3) | 9.8 (7.4-13.1) | 11.3 (8.9-14.0) | 12.7 (9.8-16.0) | <0.001 | <0.001 | <0.001 | <0.001 |
| LDL4 ApoB (mg/dl) | 13.8 (10.2-18.0) | 13.0 (9.6-17.5) | 13.4 (9.8-16.8) | 15.3 (11.6-19.3) | <0.001 | 0.889 | <0.001 | <0.001 |
| LDL5 ApoB (mg/dl) | 16.7 (12.7-21.5) | 18.3 (13.8-22.7) | 14.1 (10.7-17.7) | 16.6 (12.9-21.5) | <0.001 | <0.001 | <0.001 | <0.001 |
| LDL6 ApoB (mg/dl) | 19.8 (15.4-25.8) | 22.0 (17.0-28.5) | 15.8 (12.6-20.3) | 19.9 (16.1-25.1) | <0.001 | <0.001 | <0.001 | <0.001 |

**Supplementary Tab. S1:** Lipoprotein subclasses in the main study population and separated by sex and menopausal status. Continuous data are presented as median (25th quartile; 75th quartile). Overall group comparisons were performed using Kruskal-Wallis tests. Pairwise comparisons for men vs. premenopausal women, men vs. postmenopausal women, pre- vs. postmenopausal women were performed using Wilcoxon tests. P-values from these tests were corrected for multiple testing using the false discovery rate (FDR)

| **Characteristics** | **Main analysis** | **Sensitivity analyses** | |
| --- | --- | --- | --- |
|  | All participants  (n = 3199) | Excluding lipid- lowering medication  (n = 2824) | Excluding non- fasting participants  (n = 2004) |
| Age (years) | 52.0 (40.0 - 64.0) | 50.0 (38.0 - 61.0) | 51.0 (39.0 - 62.0) |
| Sex (% female) | 52.1 | 53.3 | 54.0 |
| Fasting time (hours) | 11.3 (2.7 - 13.4) | 11.3 (2.8 - 13.3) | 12.9 (11.6 - 14.2) |
| Smoking (%) |  |  |  |
| never smokers | 36.6 | 36.4 | 38.6 |
| former smokers | 35.9 | 34.4 | 36.8 |
| current smokers | 27.5 | 29.2 | 24.6 |
| BMI (kg/m²) | 27.5 (24.6 – 30.8) | 27.1 (24.3 – 30.6) | 27.3 (24.6 – 30.6) |
| Waist circumference (cm) | 90.2 (80.5 - 100.0) | 89.1 (79.8 - 98.8) | 89.5 (80.0 - 99.0) |
| Body fat (kg) | 22.4 (17.6 - 29.1) | 22.1 (17.3 - 28.8) | 22.4 (17.6 - 28.9) |
| HbA1c (%) | 5.20 (4.90 - 5.60) | 5.20 (4.90 - 5.60) | 5.20 (4.80 - 5.60) |
| Diabetes mellitus (%) | 10.5 | 8.11 | 6.59 |
| Arterial hypertension (%)** | 47.1 | 42.4 | 43.6 |
| Myocardial infarction (%)** | 2.22 | 0.57 | 1.65 |
| Stroke (%)** | 1.88 | 1.11 | 1.10 |
| hsCRP (mg/L) | 1.29 (0.68 - 2.86) | 1.29 (0.68 - 2.86) | 1.23 (0.67 - 2.64) |
| Total cholesterol* (mmol/L) | 5.68 (4.90 - 6.55) | 5.77 (5.00 - 6.63) | 5.81 (5.00 - 6.66) |
| LDL-cholesterol* (mmol/L) | 3.44 (2.85 - 4.10) | 3.52 (2.94 - 4.18) | 3.51 (2.89 - 4.17) |
| HDL-cholesterol* (mmol/L) | 1.50 (1.28 - 1.78) | 1.51 (1.29 - 1.80) | 1.53 (1.32 - 1.82) |
| Total triglycerides* (mmol/L) | 1.30 (0.96 - 1.80) | 1.27 (0.93 - 1.77) | 1.22 (0.90 - 1.67) |
| Dyslipidemia (%)** | 22.4 | 12.15 | 21.1 |
| Lipid-lowering MED (% yes) | 11.7 | - | 9.93 |
| eGFR (mL/min per 1.73m²) | 111.4 (99.2 - 121.0) | 113.0 (102.0 - 122.0) | 112.5 (102.0 - 121.4) |
| CKD (%) | 2.56 | 1.66 | 1.30 |
| Chemerin (ng/mL) | 98.2 (83.0 - 117.1) | 97.0 (82.2 - 115.6) | 97.4 (82.5 - 116.2) |
| Adiponectin (µg/mL) | 7.00 (4.68 - 10.20) | 7.12 (4.78 - 10.40) | 7.07 (4.74 - 10.30) |

**Supplementary Tab. S2:** Characteristics of the study populations in the main analysis and the sensitivity analyses. Continuous data are presented as median (1^st^ – 3^rd^ quartile); nominal data are given as percentages.

*Lipoproteins were measured using nuclear magnetic resonance spectroscopy. **For n=4 participants information on arterial hypertension was unavailable. For n=3 participants information on myocardial infarction or stroke was unavailable. For n=73 participants information on dyslipidemia was unavailable.

Diabetes mellitus was defined as self-reported physician’s diagnosis, intake of antidiabetic medication (ATC A10), a random glucose concentration ≥11.1 mmol/l or a HbA1c ≥6.5%. Arterial hypertension was defined as self-reported physician’s diagnosis, intake of self-reported antihypertensive medication, a systolic blood pressure ≥140 mmHg or a diastolic blood pressure ≥ 80 mmHg. Previous myocardial infarction and stroke were defined according to self-reported physician’s diagnoses. Dyslipidemia was defined as self-reported physician’s diagnosis or intake of lipid-lowering medication. Chronic kidney disease (CKD) was defined as an eGFR <60 ml/min/1.73m². BMI, body mass index; hsCRP = high-sensitivity C-reactive protein; LDL = low-density lipoprotein; HDL = high-density lipoprotein; MED = medication; eGFR = estimated glomerular filtration rate.
